# Parliament and the Profession

**Published:** 1918-07-27

**Authors:** 


### PARLIAMENT AND THE PROFESSION.

### Hospital Ships and the Mons Star.

Colonel Sir H. Greenwood asked the Under-Secretary of State for War whether he was aware that men who served on the hospital ships bringing home the wounded from France between the outbreak of war and November 22, 1914, have had the riband of the 1914 Star, which they were previously wearing, withdrawn on the ground that they were not entitled to it; and if he was aware that one of those ships served for a time between the above-mentioned dates as a base hospital, and that the men had to go ashore to carry off the wounded from the tramcars and to do picket duty. Mr. Macpherson said that it had been decided that the *personnel* of hospital ships are not entitled to the Star by virtue of the service thereon. He would, however, inquire into the special case mentioned in the question.

### Naval Surgeons.

In reference to some questions as to the conditions of service and remuneration of naval surgeons, Dr. Macnamara stated that the whole question of pay, prospects, and other matters in connection with the medical branch of the Navy is now under the consideration of the Board of Admiralty.

### Railwaymen's Convalescent Home.

Sir Willoughby Dickinson asked the Under-Secretary of State for War whether the Railwaymen's Convalescent Home at Leasowe Castle, Cheshire, which was commandeered for the purpose of training soldiers, is now occupied by German prisoners; and Mr. Macpherson said that inquiries are being made as to this circumstance of the local military authorities.

### Army Nursing Staff.

Major Chapple asked the Under-Secretary of State for War: (1) whether, in appointing nurses to the nursing staff of the Army, any discrimination is made against nurses trained in hospitals that farm out their nurses after the end of their second year's training, taking them away from their training in the wards and paying them 13s. per week while they are earning £2 2s. per week for the hospital; (2) whether the certificate given to nurses at the end of their second year's training in the London Hospital is accepted by the Army nursing authorities as qualifying for appointment to the Army nursing staff? Mr. Macpherson said that the regulations regarding the qualifications for appointment to the Queen Alexandra's Imperial Nursing Service provide that a candidate must possess a certificate of not less than three years' training and service in medical and surgical nursing in a civil hospital having not less than 100 beds. Time spent in private nursing is not allowed to count towards the qualifying period of three years' training. The answer to the second question is in the negative.

### Army Medical Establishments in France.

Major Chapple asked whether the Army Council had now considered the report of the Committee on Medical Establishments in France, and whether any action is to be taken in the matter. Mr. Macpherson stated that the report had been considered and such action as was desirable had been taken.

### Homes for Functional Nerve Disorders.

Sir A. Griffith-Boscawen informed Colonel Yate that for functional nerve disorders six homes of recovery for ex-Service men have been established. There is a home at each of the following places: London (120 beds), Leicester (60 beds), Manchester (100 beds), Edinburgh (30-60 beds), Dublin (33 beds), Belfast (62 beds). There is no connection between these homes and lunacy administration. The number of uncertifiable mental deficiency cases is not large, and where necessary arrangements have been made with one or other of the existing mental homes which accept uncertifiable cases.

### Chemists in Military Hospitals.

Mr. Forster stated that the revised rates of pay to chemists and dispensers in military hospitals are now in force and amount approximately to 15 per cent.

